# From Days to Hours: Reporting Clinically Actionable Variants from Whole Genome Sequencing

**DOI:** 10.1371/journal.pone.0086803

**Published:** 2014-02-05

**Authors:** Sumit Middha, Saurabh Baheti, Steven N. Hart, Jean-Pierre A. Kocher

**Affiliations:** Division of Biomedical Statistics and Informatics, Department of Health Sciences Research, Mayo Clinic, Rochester, Minnesota, United States of America; University of California, San Francisco, United States of America

## Abstract

As the cost of whole genome sequencing (WGS) decreases, clinical laboratories will be looking at broadly adopting this technology to screen for variants of clinical significance. To fully leverage this technology in a clinical setting, results need to be reported quickly, as the turnaround rate could potentially impact patient care. The latest sequencers can sequence a whole human genome in about 24 hours. However, depending on the computing infrastructure available, the processing of data can take several days, with the majority of computing time devoted to aligning reads to genomics regions that are to date not clinically interpretable. In an attempt to accelerate the reporting of clinically actionable variants, we have investigated the utility of a multi-step alignment algorithm focused on aligning reads and calling variants in genomic regions of clinical relevance prior to processing the remaining reads on the whole genome. This iterative workflow significantly accelerates the reporting of clinically actionable variants with no loss of accuracy when compared to genotypes obtained with the OMNI SNP platform or to variants detected with a standard workflow that combines Novoalign and GATK.

## Introduction

Whole Genome Sequencing has the potential to transform diagnostic testing in the very near future. As the cost of sequencing continues to decrease, the broader adoption of this protocol by clinical laboratories is expected. Sequencing platforms are being redesigned to accelerate the sequencing of whole genomes. For instance, the Illumina Hi-Seq 2500 platform can perform this task in 24 hours, shifting the rate-limiting step to data processing. The computationally-expensive step of aligning millions of short reads to the whole genome could be prohibitive for routine use of WGS in a clinical setting where the speed of analysis can impact patient outcome. Clinical applicability can be improved by prioritizing WGS variant reporting based on relevance for clinical decision-making. Currently, most of the clinically relevant genomics information is related to protein-coding exome regions [1] where the impact of coding variants can be interpreted in the context of proteins and their function [2], [3]. This current focus opens opportunities to develop new bioinformatics algorithms that prioritize and swiftly report clinically relevant findings.

Recently, an ultra-fast preprocessing workflow was published: ISAAC [4]. This workflow completes the whole genome alignment and variant calling in 7–8 hours. Although, ISAAC is the fastest solution currently to our knowledge, its deployment requires specific hardware and is, at least for now, limited to Illumina sequencing data.

In this manuscript we explore another approach that does not require specific hardware or software solution and is independent of the next generation sequencing platform used. Instead of expediting the whole alignment and calling process, our proposed approach prioritizes read alignment and variant calling in genomic regions of clinical relevance (referred to as the Target Reference Genome) before reporting variants in genomic regions of lower clinical significance. The proposed workflow operates in three steps. First, clinically relevant reads are selected by aligning all the sequencing data to the Target Reference Genome. Then, this reduced set of aligned reads is aligned to the whole reference genome to correct for alignment artifacts. These artifacts arise from reads forcibly aligned to the Target Reference Genome that align more accurately to non-targeted regions. After the second alignment step, reads that remain aligned on the Target Reference Genome are re-aligned and recalibrated followed by variant calling. Variants are immediately reported to clinical experts for interpretation and decision support. The final step, which can be deferred or executed at a slower pace, handles the remaining reads that are aligned on the whole reference genome.

The gain of reporting speed obtained with this iterative workflow is due to the significantly smaller size of the Target Reference Genome compared to the whole reference genome. If the targeted region corresponds to the whole exome, read alignment in the first step would be limited to less than 2% of the reference genome. Similarly, assuming even coverage, only 2% of the reads will be aligned on the whole reference genome in the second step.

Although conceptually very simple and straightforward to implement, the question of results accuracy remains to be addressed. In this manuscript, we compare results obtained by the target workflow with a generic whole genome sequencing workflow. In the process, we have also compared the impact on our iterative workflow of two aligners, BWA [5], [6] and Novoalign (http://www.novocraft.com/), on results accuracy.

## Materials and Methods

### Datasets

To test out approach, we selected a CEPH family trio from the 1000 Genomes project [7] consisting of NA12878 (Child), NA12891 (Father), and NA12892 (Mother). Each sample was sequenced using the Illumina Next Generation Sequencing Platform (HiSeq 2000) with the pair-end protocol that produced on average 397 bp long sequence fragments from which 100 bp were sequenced at both ends. Sequencing of these samples resulted in more than 2.4 billion 100 bp long reads with an average coverage of 80x across the entire genome. The Binary Alignment Map (BAM) files obtained for these samples were converted to FASTQ reads format for further analysis. The same individuals have been genotyped with a combination of Illumina and Affymetrix SNP chips for HapMap Phase III [8]. This genotype data was used to validate variants calls from sequencing data.

### Data Availability

Sequencing data: ftp://ftp-trace.ncbi.nih.gov/1000genomes/ftp/technical/working/20120117_ceu_trio_b37_decoy/.
Genotyping data: ftp://ftp.ncbi.nlm.nih.gov/hapmap/genotypes/2010-08_phaseIIIII/forward/.

### Target Reference Genome

We arbitrarily selected a set of 2638 clinically relevant genes from the Clinical Genomic Database [9]. It should be noted a smaller set similar to clinical gene panels could have been selected as well. The 2638 genes include 42048 unique exons in the UCSC RefFlat annotation. The average length of these exons is 280 bp with a standard deviation of 635 bp. The boundary of each exon was extended by 550 bp to account for the sequencing protocol that produced 100 bp long paired-end read from about 400 bp long sequence fragments. From the fragment length distribution, we estimated that an average of 0.39% read pairs would not be fully aligned with the 550 bp cutoff. The extended sequence of each exon was extracted from the Human Reference Genome (Build 37) and concatenated into a single Target Reference Genome fasta file.

### Standard sequence alignment and variant calling workflow

**Figure 1:**
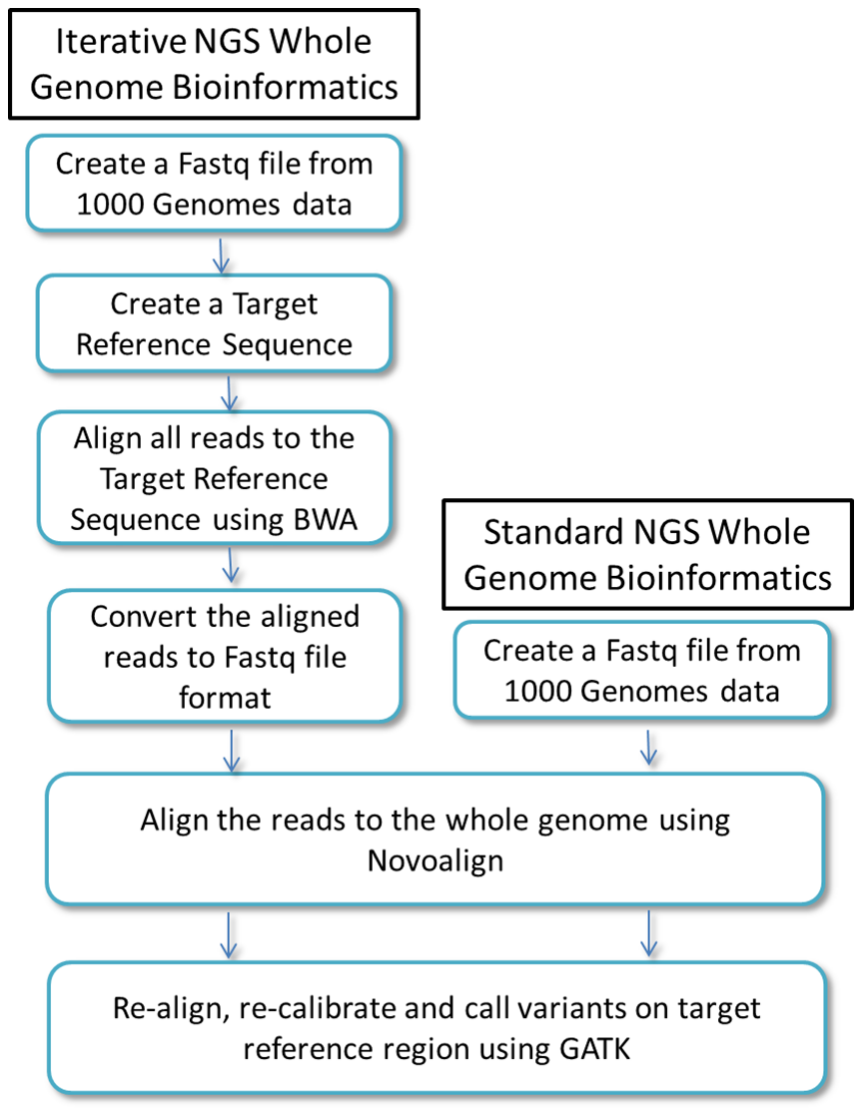
Basic components of the iterative workflow as compared to a standard NGS whole genome analysis.

As the standard whole genome alignment workflow, we used Novoalign for initial alignment of sequence reads followed by GATK for re-alignment, re-calibration and variant calling (Figure 1).

### Iterative workflow

The different steps of the iterative workflow are displayed in Figure 1. The first step filters out the reads that do not map on the Target Reference Genome while the second step refines the alignment of the mapped reads by aligning them on to the Human Reference Genome. As previously explained, this step eliminates reads that have been forcibly mapped on the Target Reference Genome but would have aligned more accurately to another location of the Human Reference Genome. Since the first alignment step produced a BAM file with mapped reads information, the BAM file was converted in FASTQ format to perform the second alignment step.

We tested the iterative workflow with two aligners BWA and Novoalign. BWA is known as being faster that Novoalign however, from our internal benchmark Novoalign produces slightly better read alignments. Since the workflow includes two alignment steps, we ran the workflow with different combinations of the two aligners. For any investigated combination of aligners, GATK was used to call variants.

## Results

The CEPH family FASTQ files were processed with both the standard and iterative workflows. The two sets of results were compared with the genotypes obtained from the OMNI SNP platform reported by 1000 Genomes project. No additional processing was done on these reported data that were used as the gold standard in this study. 18634 OMNI genotypes included in the Target Reference Genome were used for accuracy estimates.

### Results accuracy estimated from genotype calls

Using the 18634 genotypes of the OMNI SNP platform as ‘truth’, we assessed the accuracy of genotypes called by the standard workflow and the iterative workflow (Table 1). The iterative workflow results were produced with different combinations of aligners. Apart from one SNP on chromosome Y, all genotypes had adequate coverage. Results in Table 1 highlight that the BWA-Novoalign workflow has slightly higher performance accuracy than the Novoalign-Novoalign workflow. Although not necessarily significant, this result suggests that the accuracy difference that we have observed between BWA and Novoalign in the first alignment steep has little impact on the quality of the final results. However, since BWA is significantly faster than Novoalign, the BWA-Novoalign workflow completes the task more than 5 times faster than the Novoalign-Novoalign workflow. Based on these findings, our remaining analysis is limited to the results obtained with the BWA-Novoalign workflow.

**Table 1:** Concordance of SNP data with variants from standard and iterative workflows for sample NA12878.

Workflow	Aligner used in step 1	Aligner used in step 2	Number of Concordant SNVs	Number of Discordant SNVs	% Concordance	Execution time (hrs)
Standard	Novoalign	-	18344	130	99.29	73.86
Iterative	BWA	BWA	17459	947	94.88	3.09
**Iterative**	**BWA**	**Novoalign**	**18435**	**129**	**99.30**	**4.98**
Iterative	Novoalign	Novoalign	18435	129	99.30	14.09
Iterative	Novoalign	BWA	18324	172	99.07	10.03

### Genotyping calls missed by the standard and iterative workflows

About 99.3% of the genotypes called accurately by both workflows. When comparing the overlap between the 0.7% miscalled genotypes (i.e. 130 with the standard workflow and 129 with the iterative workflow), all but one of the genotypes were identical. This result reinforces the very similar performance of the two workflows and suggests that no significant bias was introduced by the iterative approach.

### Performance accuracy of iterative and standard workflows on SNVs and indels

We demonstrated that both the standard and iterative workflows had similar accuracy when compared to the OMNI SNP genotype calls. We then investigated the overlap between all the variants reported by the standard and the iterative workflow. These variants include single nucleotide variants (SNVs) and insertions/deletions (indels). The large majority of the variants were called by both workflows (Table 2). We further analyzed discordant variants not called by the two workflows. Using the basic quality metrics of quality-by-depth (QD), strand bias and low read-depth coverage of less than 10 we observed that the majority of discordant variants had poor quality. For reference, less than 3% (236 out of 8754) of the concordant SNVs had QD<5 or strand bias. We reviewed the 35 (out of 118) exclusive SNV/indel variant calls with QD>5. Out of these 35 variants, 19 have a clear strand bias. Of the remaining 16, 9 have a low coverage depth of less than 10 reads and 6 fall in a region with multiple (> = 5) homologous regions in the whole genome. This leaves just one exclusive variant of good quality that was called by our iterative workflow but not called by the standard workflow. Thus, we concluded that variants exclusively called by only one of the approaches are of low quality.

**Table 2:** Evaluation of SNVs and Indels called by the iterative and standard workflow.

Workflow	Variant	Type	NA12878	NA12891	NA12892
Iterative	SNVs	Shared	8754	8506	8809
Standard	SNVs	Shared	8754	8506	8809
Iterative	SNVs	Exclusive	38	34	39
Standard	SNVs	Exclusive	62	57	70
Iterative	INDELs	Shared	975	902	905
Standard	INDELs	Shared	975	902	905
Iterative	INDELs	Exclusive	5	5	9
Standard	INDELs	Exclusive	13	11	14

### Importance of the second alignment step

We explore the contribution of the second alignment step to the accuracy of variant calling. When using our iterative workflow, 27.5% of the reads aligned from 1st step to the CGD genes are aligned to a different location in the 2^nd^ step. When calling variants directly after the first alignment step, only 83.25% concordance is obtained with the SNP chip data compared to 99.3% concordance when the reads are processed by the second alignment step. We also observed that more than 15,000 exclusive variants are reported after the 1st alignment step, this number dropping to 100 after the second alignment step. The second alignment step in the iterative workflow is therefore critical for accurate variant calling.

### Reporting speed of clinically relevant variants

As shown in Table 1, the preferred iterative workflow takes less than 5 CPU hours to complete the alignment on the target reference genome and calling of the variants. The alignment of the remaining reads and variant calling took ∼71 CPU hours. A total of ∼76 CPU hours was therefore needed to complete the full preprocessing of the whole genome experiment. In comparison, it also took ∼76 CPU hours for the standard workflow to complete. We believe that this CPU overhead is acceptable in a clinical setup where the fast reporting of clinical variants could have a critical impact on patient's fate.

As a test, we extended Target Reference Genome to include all gene exons. The variants calls were reported in ∼15 CPU hours, still an acceptable time compared to the 76 CPU hours needed for alignment of the whole genome using standard workflow.

## Discussion

We have developed and tested an iterative whole genome sequencing workflow designed to rapidly report variants in target genomic locations. The approach first focuses on aligning all the sequence reads on the target genomic locations and then realigning this subset of mapped reads to the reference genome. We benchmarked the accuracy of the iterative workflow against genotype data used a gold standard and also compared reported SNVs to those reported by our standard whole genome sequencing workflow. Our results indicate that the standard and iterative workflows performed similarly well, with 99.3% accurate genotypes called The overlap between any variants (SNVs and Indels) called by the standard and iterative workflow is also very high (98.8%), with most of the non-concordant calls being of low confidence (low QD score).

From this analysis, we can conclude that the iterative approach does not introduce significant noise or bias that would have a negative impact on the downstream calling of variants. With regards to time, using the Target Reference Genome, which included 2638 genes, allowed for the reporting of variants called in these regions in less than 5 hours. When extending the alignment to the whole exome, results were obtained in ∼76 hours.

This iterative workflow can be particularly useful clinically when only a limited set of actionable variants need to be rapidly reported to clinicians. As compared to other published approaches, our iterative workflow does not require any additional investment in software or hardware. It is independent of the sequenced organism and the sequencing platform used as long as a reference genome is used to align the reads. Moreover, the iterative workflow can be implemented with any aligner or target reference region to swiftly report variants in those regions from whole genome sequencing data.

Finally, the third step of the alignment, which consists of aligning the remaining reads, is the most time consuming. Interestingly, in our example, these reads are now naturally organized in independent islands covering the intergenic and intronic regions of the genome, facilitating the parallel processing of read realignment in these regions. Parallelization could be a means to significantly accelerate this final step. This option, however, was not investigated in this study.
